# Novel EMD-Based Extraction Approach of Electric Field Fringe Impacts on a Micro Capacitive Displacement Sensor

**DOI:** 10.3390/mi9050211

**Published:** 2018-05-01

**Authors:** Jian-Ping Yu, Hui-Lin Du, Xin Li

**Affiliations:** 1 Key Laboratory of Air-driven Equipment Technology of Zhejiang Province, Quzhou University, Quzhou 324000, China; duhuilin@zju.edu.cn; 2 College of Mechanical Engineering, Quzhou University, Quzhou 324000, China; mexinli@zju.edu.cn

**Keywords:** capacitive micro sensor, fringe effect, co-sinusoidal error, empirical mode decomposition (EMD)

## Abstract

This paper presents an EMD (empirical mode decomposition)-based extraction approach to decouple the electric field fringe component impacts on the nonlinearity errors of a micro encoder-like capacitive displacement sensor. A calculated capacitance model built under Maxwell’s equations against the impacts of electric field fringe component indicates that signals of this sensor are all in periodic co-sinusoidal waveforms. Applying the proposed EMD scheme, signals are decomposed into sets of intrinsic mode functions (IMFs) and a residual, in which a fundamental component represents all the features of sensor signal and reserves the local information. Interpreting sensor information from the extracted component instead of the original signal drastically diminishes the impacts of electric field fringe effect. Results from a test bench shows that after applying the EMD-based extraction approach, waveform errors were decreased from over 4.18% to less than 0.89%. Nonlinearity of the interpreted displacement was decreased from 1.54% to 0.29% for 8 mm stroke.

## 1. Introduction

Capacitive sensors are probably among the most commonly used devices for micro/nano precision measurement applications, like precision bearings, fabrication of optical devices and semiconductors, organ systems, and information storages [1,2,3,4,5]. The intrinsic advantages of capacitive sensors include their fast dynamic response, proven high resolution for relatively simple structure, and effective cost [6]. Encoder-like capacitive sensors have been growing as an alternative for displacement measurement compared to conventional capacitive sensors in the last few decades, whose incremental periodic pattern makes them particularly suitable for large scale displacement measurement [6,7].

Moon et al. developed a contact-type linear high resolution encoder-like capacitive displacement sensor [8]. The sensor displayed significantly improved sensitivity without a reduction in the measurable range. Effects from certain geometrical non-idealities such as fabrication errors were considered, a compensation method was presented to minimize the effects due to non-idealities of the surface geometry of the sensor using electrode configuration. Kuijipers et al. presented a micromachined capacitive incremental position sensor, a relative displacement between the two plates resulted in a periodic change in capacitance [9]. Two comb actuators were connected along the slide path of a moving plate, the correct installation of which guaranteed the accurate measurement of the position sensor. Lee et al. presented a micro electromechanical systems capacitive position sensor for nano-positioning applications in probe storage systems [10]. A signal-processing solution was developed to compensate for the nonlinearities caused by rotational disturbances. The signal linearity was significantly enhanced, with a measured sensor signal nonlinearity of 0.78% for an 80-μm stroke. Among these sensor prototypes, different attempts were applied to diminish possible measuring errors. Nevertheless, none of them altered the fact that the electric field in reality is bent at the edges of the two plates, which is assumed as the main contributor to the nonlinearity errors of capacitive sensor.

Some solutions have been widely applied to reduce the nonlinearity errors of capacitive sensors. Equipotential electrode protection was considered one of the most feasible ways to diminish the deformation of electric field at the edge of capacitive sensor, but under the condition that two plates are completely confronted [11]. Besides this, quite a few accurate calculations of the influences of electric field fringing effect have been discovered in recent decades [12,13,14]. Benedek and Silverter presented a new method for the calculation of self and mutual capacitances of flat conductor sections [12]. The charge distribution of a capacitor was formulated in terms of a Fredholm integral equation. The resulting capacitance determination for any practical values of dielectric constant and geometric parameters was permitted to within a few percent. Wolff and Knoppik took the influence of the electrical and magnetic stray field and the influence of the fringing field into consideration as well as the influence of the inhomogeneous field distribution [13]. Using the new defined models, the accuracy for calculating the resonance frequencies was ~1%. However, these solutions were mostly built under the gap-variation capacitor model; for area-variation capacitors, when two plates moved parallel to each other, these methods hardly applied. Compared to gap-variation capacitors, the area-variation capacitors, mostly of encoder-like prototype, offer an advantage as large measuring range, are thus still limited from being widely used by the nonlinearity errors induced by electric field fringing effect.

In this work, we propose an empirical mode decomposition (EMD)-based compensation approach, which effectively decouple the electric field fringe component impacts on an encoder-like capacitive sensor. A complete description of the basic sensor structure is presented in Section 2 as well as the mathematical sensor model against fringe effect disturbances. The EMD-based filtering method is presented in Section 3. The discussion of experimental results and conclusions are proposed in Section 4 and Section 5, respectively.

## 2. Sensor Model against Fringe Effects

### 2.1. Sensor Mechanism

**Figure 1:**
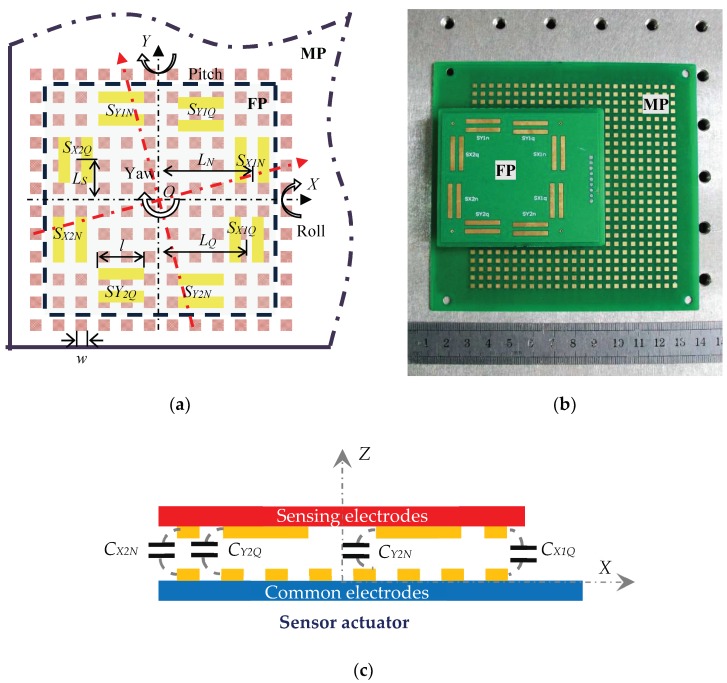
Sensor mechanism of an encoder-like capacitive positioning system. (**a**) Schematic view of a micro capacitive displacement sensor; (**b**) the actual sensor model in PCB prototype; (**c**) the capacitive incremental position sensor in concept.

The basic sensor structure of a typical encoder-like capacitive positioning system is illustrated in Figure 1a, which patterns periodic electrodes on both a moving plate (MP) and a fixed plate (FP) to maximize capacitance change. *w* is the electrode width, *l* is the sensing electrode length, *L_N_* and *L_Q_* are the distances of sensing electrodes *S_N_* and *S_Q_* from the rotational center along their measuring direction, respectively, and *L_S_* is the distance from the sensing electrodes to the rotational center in a direction perpendicular to the measuring direction.

Two pairs of normal and quadrature sensing electrodes on the FP for both X and Y displacement demodulation, which consist of eight sensing capacitors with common electrodes, namely, *S_X_*_1*N*_, *S_X_*_1*Q*_, *S_X_*_2*N*_, *S_X_*_2*Q*_, *S_Y_*_2*N*_, *S_Y_*_2*Q*_, *S_Y_*_1*N*_, and *S_Y_*_1*Q*_. Given that the common electrodes on MP are parallel patterned, capacitance variations of eight sensing capacitors are all in perfect periodic triangular waveforms when setting aside fringe effects. Noticed that four sensing capacitors (*S_X_*_1Q_, *S_X_*_2*Q*_, *S_Y_*_1*Q*_, *S_Y_*_2*Q*_) are all (1/2)*w* shifted to sensing capacitors (*S_X_*_1*N*_, *S_X_*_2*N*_, *S_Y_*_1*N*_, *S_Y_*_2*N*_) in their respective measurement directions, generating four sets of orthogonal signals. Demodulating displacement information from which would diminish the influences of roll, yaw, and pitch angular errors to the most extent.

The actual sensor model is illustrated in Figure 1b, both of the MP and the FP are of printed circuit board (PCB) prototype. A 25 × 25 square common electrode array is distributed on the MP. The length of each capacitor electrode is 2 mm. Meanwhile, 16 sensing electrodes in 8 groups are patterned on the FP, the size of which are all 2 × 16 mm.

### 2.2. Measuring Operation against Fringe Effects

Fringe effects introduce uncertain distortion on signal waveforms, which are thus the main influence of signal nonlinearity. In order to clarify these impacts, a full capacitance model calculated under Maxwell’s equations is built. Electrodes are periodically positioned on both FP and MP, therefore, when common electrodes are driven by a positive voltage *V_input_*, the potential on the surfaces of FP and MP are expressed as

(1) $$\Phi_{1}(x)=0$$

(2) $$\Phi_{2}(x)=\frac{V_{input}}{2}+\sum_{n=odd}\frac{2V_{input}}{n\pi }\sin(k_{n}x)$$

where $k_{n}=\frac{n\pi }{w}$.

The potential distribution between FP and MP can be obtained by solving the Laplace Equation by the separation of variables

(3) $$\begin{array}{l} \Phi (x,z)=A_{0}+B_{0}z+C_{0}x+D_{0}xz+\sum_{n=1}^{\infty }\left[c_{1n}\sinh(k_{n}z)\sin(k_{n}x)+c_{2n}\cosh(k_{n}z)\sin(k_{n}x\right)\\ \;+c_{3n}\sinh(k_{n}z)\cos(k_{n}x)+c_{4n}\cosh(k_{n}z)\cos(k_{n}x)] \end{array}$$

where *z* represents the displacement between the gap of FP and MP, and *A*_0_, *B*_0_, *C*_0_, *D*_0_, *c*_1*n*_, *c*_2*n*_, *c*_3*n*_ and *c*_4*n*_ are defined by the following boundary conditions

(4) $$\Phi (x,-\frac{g}{2})=\Phi_{2}(x),\;\mathrm{when}\;z=-\frac{g}{2}$$

(5) $$\Phi (x,\frac{g}{2})=\Phi_{1}(x),\;\mathrm{when}\;z=\frac{g}{2}$$

$A_{0}=\frac{V_{input}}{4}$, $B_{0}=-\frac{V_{input}}{2d}$, $C_{0}=0$, $D_{0}=0$, $c_{1n}=\frac{V_{input}}{n\pi \sinh(-k_{n}g/2)}$, $c_{2n}=\frac{V_{input}}{n\pi \cosh(-k_{n}g/2)}$, *n* = 1, 3, 5……, $c_{3n}=c_{4n}=0$.

The following equation presents the full expression of the potential between the MP and FP

(6) $$\Phi (x,z)=\frac{V_{input}}{4}-\frac{V_{input}}{2g}z+\sum_{n=odd}\left[\frac{V_{input}}{n\pi \sinh(-k_{n}g/2)}\sinh(k_{n}z)\sin(k_{n}x)+\frac{V_{input}}{n\pi \cosh(-k_{n}g/2)}\cosh(k_{n}z)\sin(k_{n}x)\right]$$

when MP travels *δ* in the measuring direction, charge distribution on which is determined by Maxwell’s equation.

(7) $$\sigma =-\varepsilon_{0}\varepsilon_{r}\frac{\partial \Phi }{\partial z}\;=-\varepsilon_{0}\varepsilon_{r}[\frac{V_{input}}{2g}+\sum_{n=odd}\frac{2V_{input}}{w}\frac{\sin(k_{n}(x-\delta ))}{\sinh(k_{n}g)}]$$

Considering the MP is grounded of the period [−*w*, 0], only the charge of the period [0, *w*] is acquired. The capacitance of the measuring capacitor can be achieved by integrating the charge distribution on the MP

(8) $$C\left(x\right)=\frac{Q}{V_{input}}=-\varepsilon_{0}\varepsilon_{r}w\left\{\frac{w}{2g}-\sum_{n=odd}\frac{4}{k_{n}w\sinh(k_{n}g)}\cos(k_{n}x)\right\}$$

**Figure 2:**
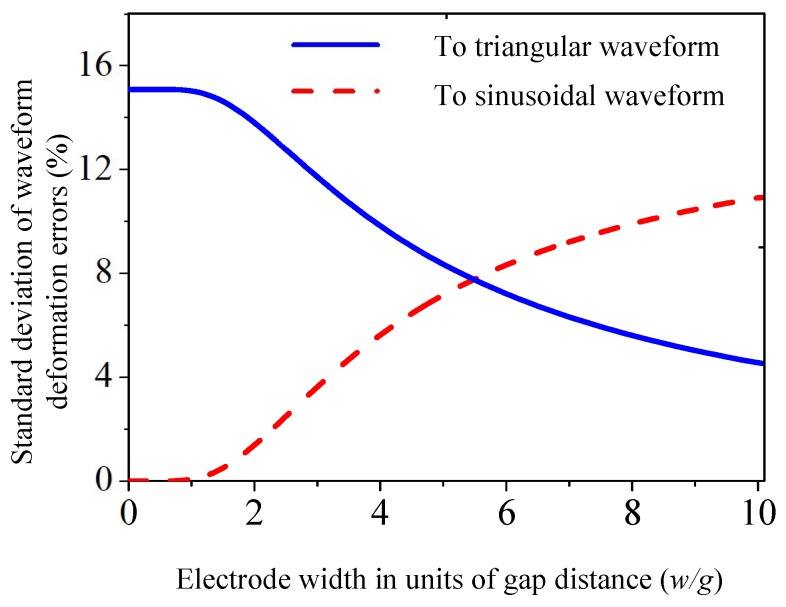
Standard deviation comparison of waveform deformation errors under different *w*/*g* ratios.

Electrode width to gap-distance ratio (*w*/*g*) is implied to be one of the key factors that determines sensor waveform. Results from Figure 2 illustrates the standard deviation comparison of waveform deformation errors, which indicates that the proposed sensor signals are neither ideal triangular nor sinusoidal waveforms. When electrode width is less than two-times the gap distance, the actual waveform is of close dependence on sinusoidal waveform, however this implementation sacrifices measuring sensitivity.

In a proposed phase shift arctangent (PSA), quadrature signal estimation method for a planar capacitive incremental displacement sensor, electrode width was set as four-times the gap distance to increase measuring sensitivity [15]. Experiment results indicated that the effects of static errors and dynamic disturbances have been mostly removed, however, waveform deformation errors remained. Certain approaches need to be applied to diminish waveform deformation errors.

Based on this conclusion, when MP and FP are completely parallel and confronted, normalized nominal output signals from the eight sensing capacitors would be in perfect sinusoidal and cosine waveforms

(9) $$\left\{ \begin{array}{l} X_{1N}^{}=\;X_{2N}^{}=\cos[2\pi \cdot (D_{X})/P]\\ -X_{1Q}^{}=X_{2Q}^{}=\sin[2\pi \cdot (D_{X})/P] \end{array}\right.$$

(10) $$\left\{ \begin{array}{l} Y_{1N}^{}=Y_{2N}^{}=\cos[2\pi \cdot (D_{Y})/P]\\ -Y_{1Q}^{}=Y_{2Q}^{}=\sin[2\pi \cdot (D_{Y})/P] \end{array}\right.$$

where *D_X_* and *D_Y_* represent the theoretical displacement of the MP in *X* and *Y* direction.

The *X*-*Y* displacement information as *D_X_* and *D_Y_* are obtained by a particular arctangent function as shown in Equations (11) and (12). Effects of some inevitable roll, yaw, and pitch angular errors would be well balanced [5,15,16].

(11) $$D_{X}=\frac{P}{2\pi }\arctan\frac{-X_{1Q}+X_{2Q}}{X_{1N}+X_{2N}}$$

(12) $$D_{Y}=\frac{P}{2\pi }\arctan\frac{-Y_{1Q}+Y_{2Q}}{Y_{1N}+Y_{2N}}$$

## 3. Theoretical Framework of EMD Filtering Method

The actual sensor signals are not in perfect sinusoidal waveforms, displacement demodulated from Equations (11) and (12) comes along with uncertain nonlinearity errors under the impacts of fringe effect. Moreover, since electrodes are patterned periodically, signal frequency is in close dependence to measuring speed. Thereby, in Section 3, for analyzing non-linear and non-stationary data, an EMD-based extraction approach is introduced.

Compared to the wavelet approach, which is usually applied in signal processing, EMD is data-driven, adaptive, and makes no assumptions about the input time-series. Any complicated data set can be adaptively decomposed into a number of IMFs. Specifically, in the case of Fourier analysis, components in a signal are defined in terms of sine and cosine waves. The EMD defines components in terms of IMFs, which are sets of symmetric mono frequency signals, or the narrow-band signals that represent the corresponding frequency components of signals [17,18,19,20].

**Figure 3:**
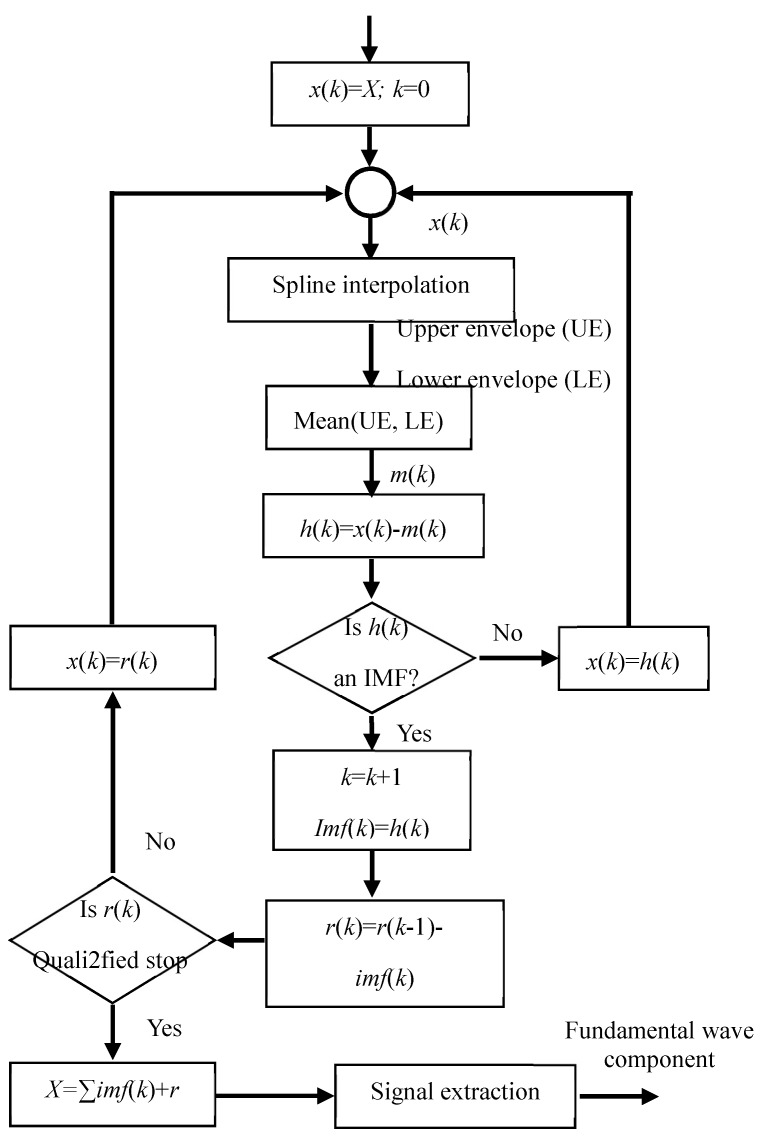
Block diagram of EMD-based extraction approach.

Specifically, EMD filtering method involves the following steps, as illustraed in Figure 3, leading to a decomposition of the signal into its constituent IMFs:

1. Define any signals from Equations (9) and (10) as *x*(*k*), where, *k* = 1.
2. Spline interpolation is fitted to acquire the lower extrema and the upper extrema, which then defines the lower (LE) and upper envelopes (UE). This approach avoids poor numerical stability due to violent oscillation, and at the same time, keeps the acquired linear interpolation continuous and smooth.
3. The average envelope, *m*(*k*), is calculated as the arithmetic mean value between UE and LE as Equation (13); where, *x_max_*(*k*) represents the upper envelope, *x_min_*(*k*) is the lower envelope.
  (13) $$m(k)=\frac{1}{2}(x_{max}(k)+x_{min}(k))$$
4. A candidate IMF, *h*(*k*), is estimated as the difference between *x*(*k*) and *m*(*k*). If *h*(*k*) satisfies the following two conditions, then an first IMF it is defined as *imf*(*k*). Otherwise, it is assigned as the new *x*(*k*) and steps 1–4 are repeated.
  (a) In the whole time-series, the number of extrema and the number of zero crossings must be either equal or differ at most by 1.
  (b) At any point in the time-series, the mean value of the upper envelope and the lower envelope is 0.
5. A partial residue, *r*(*k*) comes along with every IMF as Equation (14)
  (14) r(k)=r(k−1)−imf(k)
6. *r*(*k*) is estimated as a new *x*(*k*) and steps 1–4 are repeated until the stopping conditions—defined below—are reached, then the sifting process is finalized.
  (a) Define a maximum decomposition time, stops when residual component *r*(*k*) or the last component becomes smaller than the predetermined value.
  (b) Stops when the remaining components become monotonic functions, the intrinsic mode functions cannot be further selected.

Based on the proposed EMD scheme, signals are decomposed of sets of IMFs and a residual as in Equation (15), in which a fundamental wave component shares the same frequency with sensor signals. The fundamental component represents all the features of sensor signal and reserve the local information, thereby interpreting sensor information from the extracted component instead of the original signal drastically diminishes the impacts of electric field fringe effect

(15) $$x(k)=r(k)+\sum_{i=1}^{n}imf(i)$$

## 4. Experiments and Discussion

**Figure 4:**
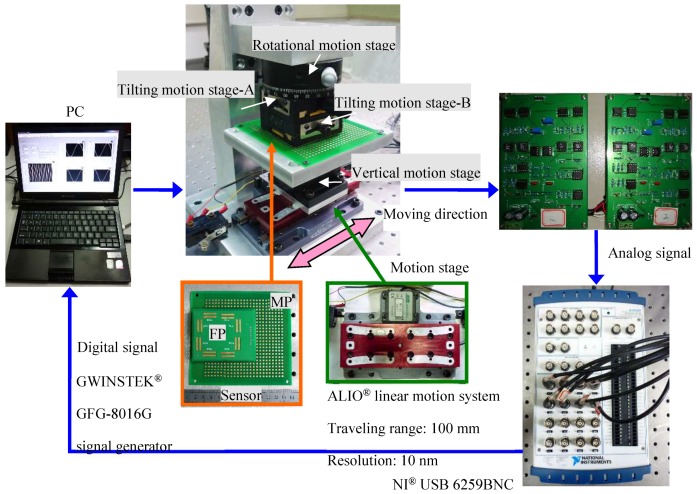
Test bench of the proposed encoder-like capacitive positioning system.

Test bench of the proposed encoder-like capacitive positioning system is designed and implemented as Figure 4, which is constituted of the PCB-based capacitive sensor, a micro motion stage, analog signal sampling circuit, and an EMD interpolation system. The test bench is implemented in a faraday cage to prevent the system from the surrounding electrostatic charges.

**Figure 5:**
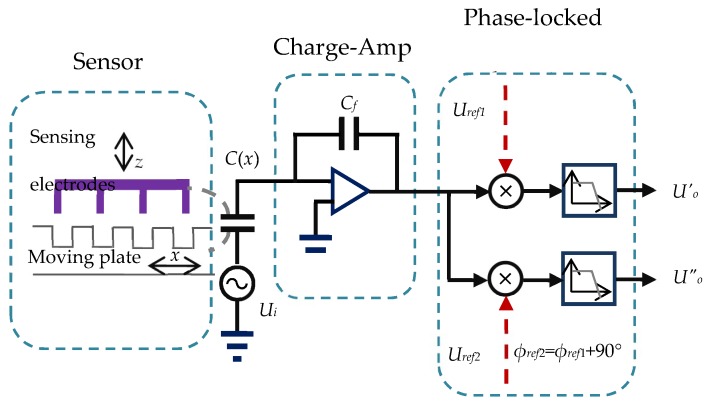
Diagram of capacitance measurement using phase-locked detection.

The MP and FP are fabricated based on a printed circuit board structure, the copper clad thickness of the MP and FP is 35 µm. The micro motion stage is consists of a NANOMOTION^®^ motor (The Johnson Electric Company, Isreal), a rotating stage, a vertical stage, and two tilting stages. The NANOMOTION^®^ motor with 10 nm resolution provides the displacement in *X* direction, the measuring speed is set as 1 mm/s. The tilting, vertical, and rotating stages are mounted for error adjustment and system calibration. The EMD interpolation system is constructed on LABVIEW^®^ (National Instruments Corporation, Austin, TX, USA), the sampling frequency of is set to 400 Hz. Phase-locked detection is applied for a signal sampling circuit as shown in Figure 5. A driving voltage with amplitude of 1 V and frequency of 1 MHz is applied at the sensing capacitors, and each resulting current is converted to a voltage by the charge amplifier (AD633, Analog Devices, Norwood, MA, USA). Two reference signals (e.g., *U_ref_*_1_ and *U_ref_*_2_) are mixed with each acquired signal, generating two output voltages as *U_o_′* and *U_o_″*. *U_ref_*_2_ shares the same frequency with *U_ref_*_1_, but whose phase position is 90° shifted to the later one. This difference is also delivered to output voltages *U_o_′* and *U_o_″*. As a result, with the use of the following equation, it is possible to eliminate parasitic capacitance changes due to changes in relative humidity, temperature, and pressure [21,22].

(16) $$U_{o}(x)=\sqrt{U_{o}^{\prime }{\left(x\right)}^{2}+U_{o}^{\prime \prime }{\left(x\right)}^{2}}$$

Of the proposed encoder-like capacitive positioning system, dc voltage signals are obtained by the signal acquisition system which are proportional to the capacitances. Instead of demodulating the specific capacitance value, the voltage signals are normalized and compared with the ideal sinusoidal waveform. The reason for this arrangement is that the measured displacements are closely related to the specific phase position of the output waveform but not to whose amplitude value. The measurement accuracy of the displacement is determined by the error distribution of the output signal to sinusoidal waveforms, which does not require specific capacitance value of the proposed sensor system.

**Figure 6:**
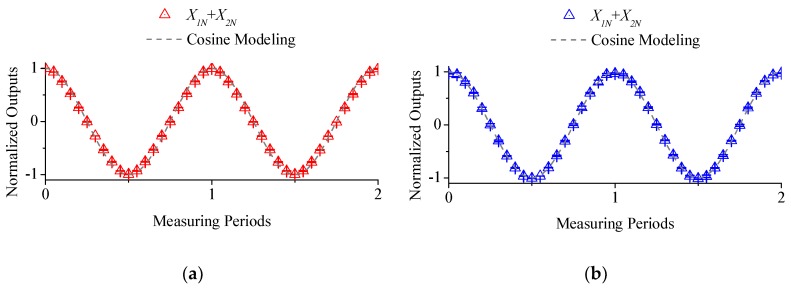
Demodulated sensor signal in *X* direction: (**a**) normalized outputs; (**b**) fundamental wave component of an EMD-based extraction approach.

**Figure 7:**
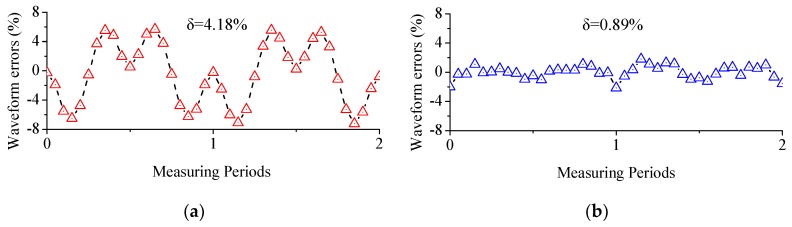
Error distribution of the demodulated sensor signal: (**a**) normalized outputs; (**b**) fundamental wave component of an EMD-based extraction approach.

Figure 6a depicts demodulated actual sensor signal in X direction when the electrode length is four times the gap distance. Specifically, gap distance is set to 0.5 mm compared to electrode length as 2 mm. The results indicates that the normalized outputs are in close dependence to cosine waveforms. However, as it shown in Figure 7a, the standard deviation of waveform errors to ideal cosine waveform are as high as 4.43%, thereby introducing severe nonlinearity errors to output waveforms.

To diminish the influences of fringe impacts, the proposed EMD-based extraction approach is applied. Signals are decomposed of sets of intrinsic mode functions (IMFs) and a residual. The IMF that shares the same frequency with the original signal is defined as the fundamental wave component. Sensor information is interpreted from the IMF instead of the original signal.

Figure 6b depicts the extracted fundamental wave component of this EMD-based demodulated sensor signal, which shares the same frequency as the original sensor signals. However, as the results in Figure 7b reveals that the standard deviation is decreased from over 4.18% down to 0.89%, waveform errors are drastically diminished. The experiments showed unanimous results to model-based fitting.

**Figure 8:**
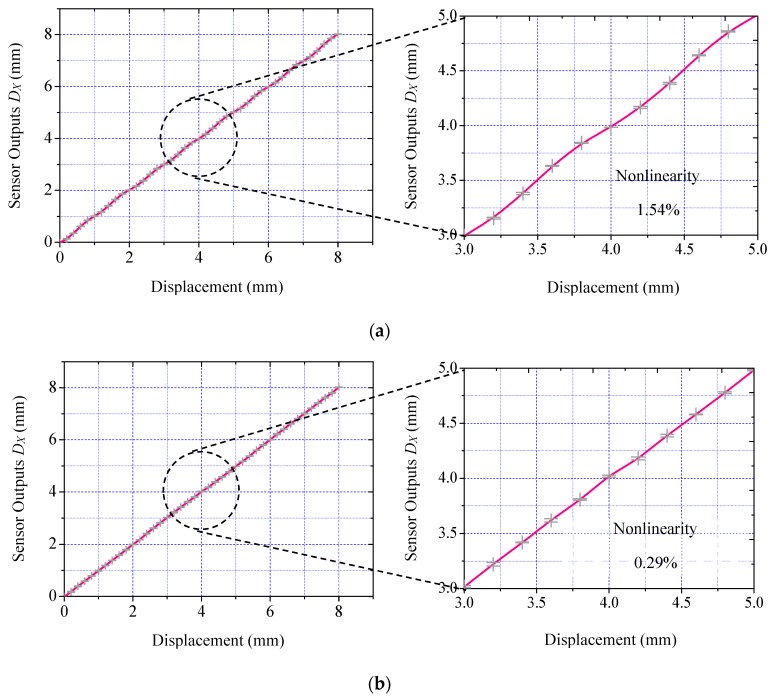
Acquired nominal displacement in *X* direction: (**a**) the conventional scheme; (**b**) the proposed scheme.

Figure 8a plots the acquired nominal displacement in X direction interpreted from the original signals. Acquired waveforms exhibit severe distortion. Signal nonlinearity is as high as 1.54%. The proposed EMD approach removes the influences of fringe effects. Figure 8b shows the acquired nominal displacement in the *X* direction by loading the EMD approach. Signal nonlinearity stays consistent as only 0.29%. This result validates effectiveness of the proposed method.

## 5. Conclusions

The development of novel EMD-based extraction approach for a micro capacitive displacement sensor is presented in this work. The capacitance model against fringe effects is established in theory, based on which, the EMD approach acquires a fundamental component that represents all the features of sensor signals. Nonliearity errors induced by fringe effect are remarkably diminished. Experimental results indicates that the estimated error distribution is decreased from 4.18% down to 0.89%. Nonlinearity of the interpreted displacement was decreased from 1.54% to 0.29% for an 8 mm stroke. Advantages of the EMD-based extraction approach interpolation method are validated.
